# Draft Genome Sequence of Enteropathogenic *Escherichia coli*, Isolated from the Bloody Stool Sample of a Common Marmoset (*Callithrix jacchus*)

**DOI:** 10.1128/genomeA.01161-15

**Published:** 2015-10-08

**Authors:** Nobuhito Hayashimoto, Hanako Morita, Takashi Inoue, Masahiko Yasuda, Masafumi Yamamoto, Toshio Itoh

**Affiliations:** a ICLAS Monitoring Center, Central Institute for Experimental Animals, Kanagawa, Japan; b Marmoset Research Department, Central Institute for Experimental Animals, Kanagawa, Japan; c Pathological Analysis Center, Central Institute for Experimental Animals, Kanagawa, Japan

## Abstract

Here, we report the draft genome sequence of *Escherichia coli* strain R811. This bacterium was isolated from the bloody stool sample of a common marmoset, and was categorized as enteropathogenic *E. coli* because it possessed *eae*.

## GENOME ANNOUNCEMENT

Enteropathogenic *Escherichia coli* (EPEC) strains are important causative pathogens of infantile diarrhea in developing countries (1). EPEC strains are also enteropathogens in common marmosets (Callithrix jacchus). However, very few studies have investigated the pathogenicity and characterization of EPEC strains in common marmosets (2).

The main virulence factor of EPEC is the ability to produce attaching and effacing (A/E) lesions (3). A/E lesions are characterized by tight attachment of the bacteria to the enterocyte surface, effacement of brush border microvilli, and formation of an actin-rich pedestal beneath the attached bacteria (2). An outer membrane protein called intimin, encoded by *eae*, mediates intestinal cell attachment. The *eae* is currently used for the molecular diagnosis of EPEC. All of the genetic elements required for the A/E lesions are encoded on a large genomic pathogenicity island called the locus of enterocyte effacement (LEE). The LEE pathogenicity island plays an important role in bacterial adherence to host intestinal epithelial cells (3). In order to study the genetic features of EPEC strains, especially those involved in pathogenicity, we sequenced a representative strain, R811, which was derived from the bloody stool sample of a common marmoset. Strain R811 was categorized as EPEC based on the positive PCR amplification of *eae* (4) and the absence of *VT1*, *VT2*, *ST*, and *LT*, which were determined with commercially available PCR kits (PowerCheck Diarrheal *E. coli* 4-plex Detection Kit; Kogenebiotech Co., Seoul, Korea).

The draft genome of *Escherichia coli* R811 was obtained by using an Illumina HiSeq 2500 sequencer (Illumina, San Diego, CA, USA), with sequencing runs for paired-end sequences. The bacterial DNA libraries were prepared with a TrueSeq DNA sample prep kit (Illumina). The genome was assembled into 386 contigs, ranging in size from 189 to 346,100 bp, by using *de novo* sequence assembler software (Velvet, EMBL-EBI) (5). Gene prediction was performed with the Rapid Annotation using Subsystem Technology server (http://rast.nmpdr.org) (6) and the Microbial Genome Annotation Pipeline (http://www.migap.org) (7). Sequencing and read assembly of the libraries were carried out by Hokkaido System Science (Sapporo, Japan).

The draft genome sequence of *E. coli* R811 was 5,444,756 bp (5.4 Mb) with a G+C content of 50.5%. The sequenced strain encoded, on average, 5,525 protein-coding genes, 7 rRNA genes, and 97 tRNA genes. Our analysis revealed several genes related to pathogenicity, including intimin (*eaeA*), RTX toxin (*rtxA1*), hemolysin E (*ClyA*), and *Shigella* enterotoxin 2 (*shET2*). Except for intimin, the other virulence factors listed here were identified for the first time in the strain in this study.

### Nucleotide sequence accession numbers.

The draft genome sequences for the *E. coli* R811 strain have been deposited in DDBJ/EMBL/GenBank under the accession numbers BBYP01000001 to BBYP01000539. The version described in this paper is the first version.
